# Correction: Askoura et al. Alteration of *Salmonella enterica* Virulence and Host Pathogenesis through Targeting *sdiA* by Using the CRISPR-Cas9 System. *Microorganisms* 2021, *9*, 2564

**DOI:** 10.3390/microorganisms14010245

**Published:** 2026-01-21

**Authors:** Momen Askoura, Ahmad J. Almalki, Amr S. Abu Lila, Khaled Almansour, Farhan Alshammari, El-Sayed Khafagy, Tarek S. Ibrahim, Wael A. H. Hegazy

**Affiliations:** 1 Department of Microbiology and Immunology, Faculty of Pharmacy, Zagazig University, Zagazig 44519, Egypt; 2 Department of Pharmaceutical Chemistry, Faculty of Pharmacy, King Abdulaziz University, Jeddah 21589, Saudi Arabia; ajalmalki@kau.edu.sa (A.J.A.); tmabrahem@kau.edu.sa (T.S.I.); 3 Center of Excellence for Drug Research and Pharmaceutical Industries, King Abdulaziz University, Jeddah 21589, Saudi Arabia; 4 Department of Pharmaceutics and Industrial Pharmacy, Faculty of Pharmacy, Zagazig University, Zagazig 44519, Egypt; a.abulila@uoh.edu.sa; 5 Department of Pharmaceutics, College of Pharmacy, University of Hail, Hail 81442, Saudi Arabia; kh.almansour@uoh.edu.sa (K.A.); frh.alshammari@uoh.edu.sa (F.A.); 6 Department of Pharmaceutics, College of Pharmacy, Prince Sattam Bin Abdulaziz University, Al-kharj 11942, Saudi Arabia; e.khafagy@psau.edu.sa; 7 Department of Pharmaceutics and Industrial Pharmacy, Faculty of Pharmacy, Suez Canal University, Ismailia 41552, Egypt

In the original publication, there was a mistake in Figure 7B as published [1]. The microscope image of the translocation of SPI2-TTSS effector proteins in macrophages was inserted incorrectly. The corrected Figure 7 appears below. 


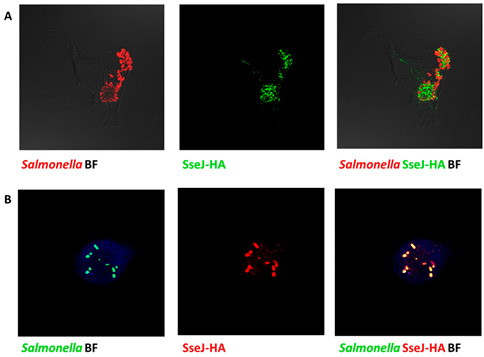



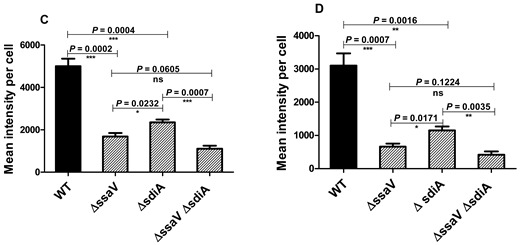


The authors state that the scientific conclusions are unaffected. This correction was approved by the Academic Editor. The original publication has also been updated.
